# A hydrodynamics assessment of the hammerhead shark cephalofoil

**DOI:** 10.1038/s41598-020-71472-2

**Published:** 2020-09-02

**Authors:** Matthew K. Gaylord, Eric L. Blades, Glenn R. Parsons

**Affiliations:** 1 Department of Biology, Center for Biodiversity and Conservation Research, The University of Mississippi, University, MS USA; 2 Center for Advanced Vehicular Systems, Mississippi State University, Mississippi State, MS USA; 3 Present Address: ATA Engineering, Huntsville, AL USA

**Keywords:** Physiology, Engineering

## Abstract

Hammerhead sharks are characterized by a conspicuous lateral expansion of the head forming a structure known as a cephalofoil. Two theories regarding the function of this structure suggest that it may increase maneuverability as well as produce dynamic lift similar to a cambered airplane wing. Here we report on a family-wide computational fluid dynamics analysis of all eight hammerhead shark species and three sharks with typical head shape. Models cast of the heads of fresh and museum specimens of hammerhead and typical sharks were used to produce pressure surface maps and lift and drag polar diagrams at various angles of attack. These analyses suggested that the cephalofoil (1) provides greater maneuverability that may be important in prey capture efficacy, (2) does not provide significant dynamic lift when held parallel to flow, (3) is characterized by greater drag than typical sharks across all attack angles, and (4) was found to result in a 10-x increase in energetic cost over typical shark head morphologies.

## Introduction

Hammerhead sharks (family: Sphyrnidae) are a common, widely distributed group composed of eight extant species constituting two separate genera (*Sphyrna* and *Eusphyra*). Of these, seven belong to the genus *Sphyrna*, whereas the genus *Eusphyra* is represented by only a single species, the winghead shark (*E. blochii*). Hammerhead cranial morphologies are united by strong dorsoventral flattening and lateral expansion resulting in an anterior cephalofoil or “head-wing” with the eyes situated distally at each lateral end; however, substantial morphological variation exists across the clade.

The function of the cephalofoil has historically been the subject of much conjecture. In short, three general theories have been advanced to ascribe adaptive significance to the sphyrnid cephalofoil. (1) The structure has been hypothesized to provide sensory advantages by increasing olfactory, visual, and/or electrosensory abilities^1,2^. (2) More recently, observations of hammerhead shark behavior suggested that the cephalofoil may increase prey handling capabilities^3^. (3) The cephalofoil may serve a hydrodynamic function by increasing maneuverability and/or providing hydrodynamic lift^4–6^. This paper addresses the third theory.

Because they lack a swim bladder, elasmobranchs (sharks, skates and rays) must rely on other mechanisms for buoyancy regulation. One long-standing supposition (often stated as fact) is that the cephalofoil acts as a “wing” producing lift forces that aid the shark in maintaining vertical station in the water column^4,5^. Indeed, in both profile and parasagittal section, each lobe of the cephalofoil resembles a cambered wing^7^. A cambered wing is more convex on the dorsal surface, relative to ventral, which causes air or water to accelerate as it passes across the dorsal surface. The increase in speed results in lowered pressure on the dorsal surface and lift can be produced even when the angle at which the wing is held relative to flow is zero. This contrasts with a symmetrical wing that produces no lift at zero attack angle. In spite of nearly a century of speculation, empirical data regarding hydrodynamic properties of the cephalofoil are strikingly scarce.

Computational fluid dynamic (CFD) methods, although common within the engineering field, are largely underutilized within the biological sciences. To our knowledge, only two papers have been published that used CFD to examine vertebrate swimming^8,9^. The unique advantage of CFD is that it affords researchers a virtual study environment that is largely free of the typical sources of extraneous variation. Moreover, CFD is cost-effective, its models are easy to manipulate, and it enables flow visualization at levels of resolution otherwise impossible to obtain in a conventional laboratory setting.

Two primary mechanisms have been proposed regarding hydrodynamic function of the cephalofoil. The first of these suggests that it increases maneuverability by acting as an anterior steering wing^10^. In the second mechanism, the cephalofoil functions as a planing surface, contributing to the shark’s achievement of neutral buoyancy by acting as a hydrofoil to generate lift^4,5^. To date, only one study^11^ has sought to directly evaluate the hydrodynamic attributes of the cephalofoil and its capacity to generate lift. In that study, lift-to-drag (L/D) ratios for model representations of the cephalofoil of eight hammerhead species were determined using a wind tunnel. While results of that study suggested that the cephalofoil produces lift, data indicated that airfoil behavior was limited to high Reynolds numbers (i.e., Re > 4.2 × 10^5^), and models were sculpted by hand. Vortex shedding was examined using a flow tank, but again, head models were handmade, and experimentation was carried out over a limited range of angles of attack.

In this report, we used CFD flow simulation to investigate the idea that the cephalofoil may provide an increase in maneuverability, that it may provide hydrodynamic lift similar to that of a cambered wing, and calculated the drag and energetic cost of possessing a cephalofoil.

## Materials and methods

### Model description

Shark specimens for CFD analysis were obtained from a variety of sources. Two-part molds were used to cast plaster models of the heads of all eight extant species of hammerhead shark (*E. blochii, S. corona, S. lewini, S. media, S. mokarran, S. tiburo, S. tudes, and S. zygaena*) as well as three carcharhinid species (*C. leucas, C. limbatus,* and *N. brevirostris*). Head models were digitized using a Faro-Arm laser scanner at Mississippi State University’s Center for Advanced Vehicular Systems (CAVS), and resulting point cloud data were imported using Geomagic Studio 10 software. Models were rotated and rough-translated so as to properly orient them within the world coordinate system (the coordinate system that is defined in our flow solver, U^2^NCLE^12^, and serves to locate a measurement in some multi-dimensional parameter space). NURBS models were created from polygon data by using the *fit surface* feature before exportation as IGES files. Blender V2.49b was used to scale all models to adult size and to fine-translate models against the world coordinate system grid. To accomplish this, scaling factors were calculated based on a composite data set compiled from published study material and firsthand specimen observations. Each head was oriented in Blender’s *side view* and *top view* before importation into SolidMesh V3.2.2 grid generation software^13^. For each model, a generic, conical body shape was created in SolidMesh by projecting the posterior edge onto an *x–y* plane created at approximately three to four times the total head length in the negative *z*-direction. A conical shape was chosen so as to facilitate gradual flow separation toward the posterior end of the heads (rather than unrealistically abrupt separation which would contribute drastically to form drag). A spherical farfield boundary defining the outer limit of the fluid domain was created for each model. The radius of the farfield boundary was approximately ten times the total length of the model, and the farfield boundary was assigned an inviscid boundary condition. Finally, all edges were glued to yield topologically correct surfaces.

**Figure 1:**
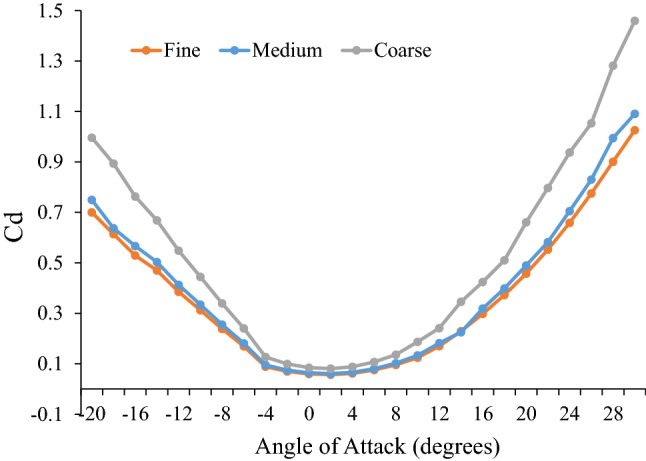
Results of the mesh refinement study for *S. corona.* Coefficient of drag estimated for fine, medium and coarse levels of mesh refinement.

SolidMesh^12^ was used to generate unstructured, triangular surface meshes of each model. Point spacings, which define the local mesh size at discrete locations on the model and are used to control or specify the fidelity of the surface mesh resolution, were assigned to the models and manually refined in regions of high curvature (i.e., for sphyrnids, the leading and trailing edges of the cephalofoil; for carcharhinids, the tip and edges of the rostrum). For one species (*S. corona*) a mesh refinement study was conducted to assess the adequacy of mesh resolution (Fig. 1). Three separate levels were generated (referred to as *coarse, medium,* and *fine*) and with each successive level of mesh refinement, model point spacings were reduced by a factor of 2.0, thus increasing the spatial resolution of the surface mesh. Note that the volume mesh generator used in this work, AFRL3^14^, uses an advancing front algorithm and therefore the resolution of the surface directly affects the spatial resolution of the volume mesh, i.e. increasing the surface mesh resolution also increases the resulting volume mesh resolution. Both *C. leucas* and *N. brevirostris* were modeled at the medium level of grid fineness due to difficulties encountered with quality in some regions of the grid. SolidMesh^12^ was used in concert with the Advancing-Front Local Reconnection (AFLR3) volume mesh generation software^13,14^ to discretize the fluid domain of each species model using a mixed-element mesh (Table 1). Estimates of freestream fluid velocity were calculated using the equation for fish optimum cruising speed:

**Table 1:** Summary of Reynolds numbers, boundary layer element initial spacing (m), and total mesh nodes for volume meshes of each species.

Species	Reynolds number	Initial spacing	Nodes
*Eusphyra blochii*	3.61 × 10^4^	0.00249994	2,672,080
*Sphyrna corona*	3.74 × 10^4^	0.00315047	1,449,337
*Sphyrna lewini*	1.25 × 10^5^	0.00215024	11,866,016
*Sphyrna media*	4.23 × 10^4^	0.00275701	1,300,115
*Sphyrna mokarran*	1.24 × 10^5^	0.00210281	10,308,942
*Sphyrna tiburo*	4.04 × 10^4^	0.00279164	1,114,575
*Sphyrna tudes*	5.99 × 10^4^	0.00270792	3,018,520
*Sphyrna zygaena*	1.14 × 10^5^	0.00258688	4,931,330
*Carcharhinus leucas*	1.95 × 10^5^	0.00219776	4,852,148
*Carcharhinus limbatus*	1.32 × 10^5^	0.00264026	8,267,361
*Negaprion brevirostris*	1.91 × 10^5^	0.00205260	4,166,873

$$U_{0} = {\text{ 6}}.{\text{3}}L_{T} ^{{0.{\text{43}}}}$$

where *L*_*T*_ is fish total length (in cm) and *U*_*0*_ is freestream velocity (cm/s)^15^.

Scale factors (reference lengths) were calculated for each species by measuring the chord at twelve locations spaced evenly across the head (in the *x*-dimension) and taking the average of these.

Reynolds numbers were calculated for each species and defined as:

$${Re = \frac{U \cdot L}{v}}$$

where *U* is the freestream velocity (m/s), *L* is the reference length (m), and *v* is the kinematic viscosity of seawater (9.37 × 10^−7^ m^2^/s) at 25 °C and 35ppt salinity.

The y^+^ value is a non-dimensional wall distance that serves as a measure of boundary layer resolution. It is typically desirable to use an initial spacing that results in an actual y^+^ of approximately 1.0 at the first point off the viscous wall boundary. The y^+^ value for all models was set to 0.8 (to compensate somewhat for curvature of the model as automatic settings in SolidMesh are calculated based upon a *flat plate* reference geometry). Anisotropic element initial spacing was set for each model automatically in SolidMesh based upon *Re*, Y^+^, and *L.* The maximum growth rate factor (1.38819) for isotropic cells (outside of the anisotropic boundary layer) was likewise set automatically.

### CFD simulations

Simulations were performed using the U^2^NCLE^16^ unstructured flow solver, a parallel flow computational fluid dynamics code developed at Mississippi State University’s Center for Advanced Vehicular Studies. U^2^NCLE is a parallel flow simulation code that solves the Unsteady Reynolds-Averaged Navier–Stokes equations for complex geometries represented by multi-element unstructured grids. U^2^NCLE is capable of solving inviscid, laminar, and high Reynolds number flows for either steady or unsteady conditions. An overview of the U^2^NCLE flow solver and the numerical algorithms it employs is included here.

### Governing equations

The unsteady three-dimensional incompressible Reynolds-averaged Navier–Stokes equations are presented here in Cartesian coordinates and in conservative form. An artificial time derivative term (${{\partial \rho_{\alpha } } \mathord{\left/ {\vphantom {{\partial \rho_{\alpha } } {\partial t}}} \right. \kern-\nulldelimiterspace} {\partial t}}$, where $\rho_{\alpha } = {P \mathord{\left/ {\vphantom {P \beta }} \right. \kern-\nulldelimiterspace} \beta }$) has been added to the continuity equation to cast the complete set of governing equations into a time-marching form^17^. The non-dimensional equations can be written in integral form as

1 $$\frac{\partial }{\partial t}\int_{\,\Omega } Q \,dV\, + \,\int_{\,\partial \Omega } { \, \overrightarrow {F} } \,.\,\hat{n}\,dA\,\, = \,\,\frac{1}{{\text{Re}}}\int_{\,\partial \Omega } { \, \overrightarrow {G} } \,.\,\hat{n}\,dA$$

The vector of dependent variables and the components of the inviscid and viscous fluxes are given as

2 $$Q\,\, = \,\,\left[ {\begin{array}{*{20}c} P \\ u \\ v \\ w \\ \end{array} } \right]{, }\vec{F}.\hat{n}\,\, = \,\,\left[ {\begin{array}{*{20}c} {\beta V} \\ {u\Theta + \hat{n}x\,P} \\ {v\Theta + \hat{n}y\,P} \\ {w\Theta + \hat{n}z\,P} \\ \end{array} } \right],\,\,\vec{G}\, \cdot \,\hat{n}\, = \,\left[ {\begin{array}{*{20}c} 0 \\ {\hat{n}x\,\tau xx + \hat{n}y\,\tau xy\, + \,\hat{n}z\,\tau xz} \\ {\hat{n}x\,\tau yx + \hat{n}y\,\tau yy\, + \,\hat{n}z\,\tau yz} \\ {\hat{n}x\,\tau zx + \hat{n}y\,\tau zy\, + \,\hat{n}z\,\tau zz} \\ \end{array} } \right]$$

The velocity normal to a control volume face is defined as

3 $$\Theta = \hat{n}xu + \hat{n}yu + \hat{n}zu + at$$

and the grid speed is defined as

4 $$a_{t} = - \left( {V{x} \hat{n}_{x} + V{y} \hat{n}_{y} + V{z} \hat{n}_{z} } \right)$$

Note that $\overrightarrow {V}{s} = V{x} \hat{i} + V{y} \hat{j} + V{z} \hat{k}$ is the control volume face velocity. The artificial compressibility factor ($\beta$) is typically set to 15. The variables in the preceding equations are non-dimensionalized using a characteristic length (L), and free-stream values of velocity $\left( {U_{\infty } } \right)$, and kinematic viscosity $\left( {\upsilon_{\infty } = {{\mu_{\infty } } \mathord{\left/ {\vphantom {{\mu_{\infty } } {\rho_{\infty } }}} \right. \kern-\nulldelimiterspace} {\rho_{\infty } }}} \right)$. Thus, Reynolds number is defined as $\frac{U\infty L}{{\nu \infty }}$ and pressure is normalized by $\frac{{P^{*} - P_{\infty } }}{{\rho_{\infty } U_{\infty }^{2} }}$, where $P^{*}$ is the local dimensional static pressure and $\rho_{\infty }$ is the free-stream density. The viscous stresses are $\tau ij = \left( {\mu + \mu t} \right)\,\left( {\frac{\partial ui}{{\partial xj}} + \frac{\partial uj}{{\partial xi}}} \right)$.

### Solution algorithm details

The U^2^NCLE flow solver is a node-centered, finite volume, implicit scheme applied to general unstructured grids with nonsimplical elements. The flow variables are stored at the vertices and surface integrals are evaluated on the median dual surrounding each of these vertices. The non-overlapping control volumes formed by the median dual completely cover the domain, and form a mesh that is dual to the elemental grid. Thus, a one-to-one mapping exists between the edges of the original grid and the faces of the control volumes.

The solution algorithm consists of the following basic steps: reconstruction of the solution states at the control volume faces, evaluation of the flux integrals for each control volume, and the evolution of the solution in each control volume in time. The inviscid fluxes are computed using the flux-difference splitting technique^18^. A spatially 2nd-order accurate method is used for the inviscid flux terms where the primitive variables $\vec{q}^{T} = \left[ {\rho ,u,v,w,p} \right]$ are extrapolated to the faces of the control volume using a second-order Taylor series expansion about the central vertex. The linear reconstruction at the control volume face is given by $\vec{q}_{f} = \vec{q}_{0} + \nabla \vec{q}_{0} \cdot \vec{r}$ where $\vec{q}_{f}$ is the reconstructed function, $\nabla \vec{q}_{0}$ is the gradient of primitive variables at the vertex, and $\vec{r}$ is the vector from the vertex to the midpoint of the edge, and a least squares approximation is used to construct the gradient. Barth’s limiter^19^ is also applied to these terms to prevent overshoots and undershoots. The viscous flux vector, $\overrightarrow{G}\left(Q\right)$ in (Eq. 2), represents the shear stress effects and contains terms involving velocity gradients. A finite volume technique with a direct approximation for the gradients at the quadrature points is used to discretize the viscous contributions. The viscous fluxes are evaluated directly at each edge midpoint using separate approximations for the normal and tangential components of the gradient vector to construct the gradients required in the viscous fluxes. The temporal discretization for unsteady simulations is done using a 2nd-order in time backward Euler implicit scheme and a first order backward Euler scheme is used for steady-state simulations. The time evolution is accomplished using a Newton relaxation scheme to advance the unsteady solution at each time step^20^. The one-equation turbulence model of Spalart and Allmaras was used for simulation of turbulent effects in high Reynolds number flow^21^. The authors of the original Spalart–Allmaras turbulence model presented results for external flows with attached boundary layers and flows with mild separation which are similar to the type of flows encountered in this work, and it is these types of flows for which the model will yield the best results. Steady, RANS solutions were computed to predict the flow field and force and moment coefficients for adults of all species across twenty-six different angles of attack.

Flow was modeled in the negative *z*-direction, and coordinates for *fluid direction* and *lift direction* vectors were calculated for all as,

$${\text{Fluid}}\;{\text{direction}}:x = 0,\;y = sin\left( \alpha \right),\;z = - cos\left( \alpha \right)$$

$${\text{Lift}}\;{\text{direction}}:x = 0,\;y = - cos\left( \alpha \right),\;z = sin\left( \alpha \right)$$

where α is the deflection angle (in degrees) with respect to freestream flow.

## Results

**Figure 2:**
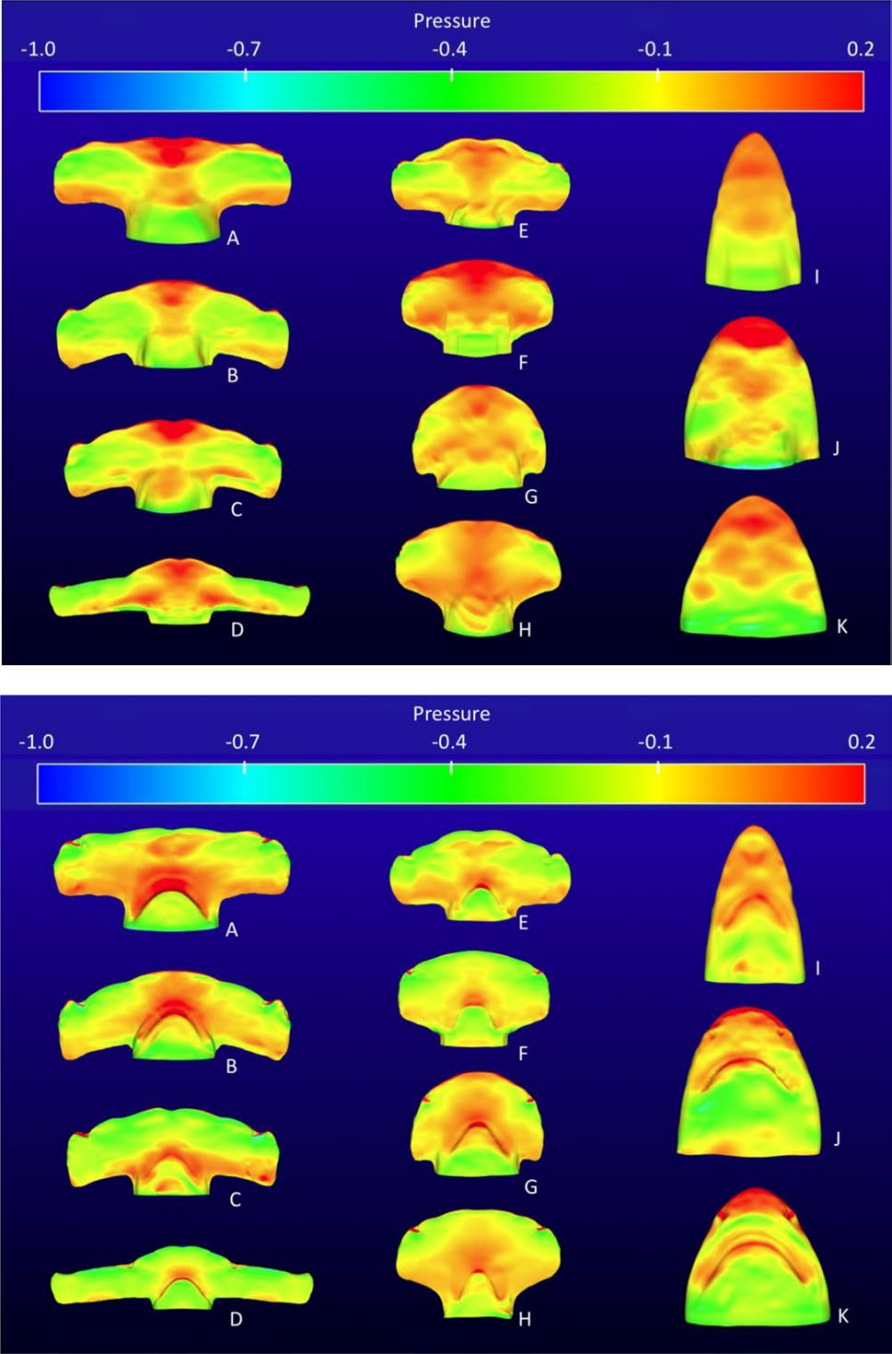
Composite of *dorsal* (top) and v*entral* (bottom) surface pressure contours from all eleven species at zero angle of attack. Note the similarity in magnitude (both interfamilial and between dorsal and ventral surface pressure contours); (**A**) *S. mokarran*, (**B**) *S. zygaena*, (**C**) *S. lewini*, (**D**) *E. blochii*, (**E**) *S. tudes*, (**F**) *S. media,* (**G**) *S. tiburo,* (**H**) *S. corona*, (**I**) *C. limbatus,* (**J**) *C. leucas*, (**K**) *N. brevirostris.*

Qualitative comparison of pressure isosurfaces indicated similar pressure magnitudes at α = 0°, over both dorsal and ventral (Fig. 2) surfaces for all specimens. Pressure contours depicted the highest pressures across the dorsal surfaces near the central leading edge of the cephalofoil and just ahead of the mouth on the ventral surfaces. Large areas of low pressure were evident on both the dorsal and ventral surfaces of the cephalofoil (Fig. 2).

**Figure 3:**
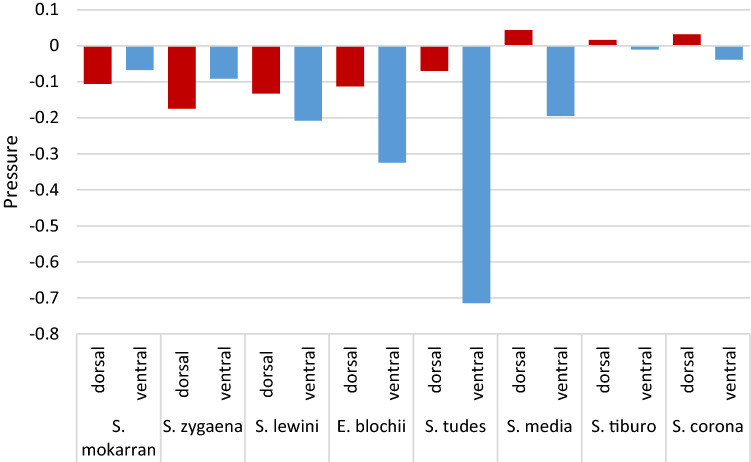
Area-weighted averages of pressures across the *dorsal* and *ventral* surfaces of the cephalofoil at zero angle of attack for each hammerhead species.

We used area-weighted averages to quantify pressures across the dorsal and ventral surfaces of each hammerhead species (Fig. 3). In five of the eight hammerhead species, both dorsal and ventral pressures were less than zero. Mean dorsal pressures were higher than ventral pressures in six of the eight species (*S. lewini, tudes, media, tiburo* and *corona,* and *E. blochii*) and, in most cases, dorsal pressures were much higher than ventral. Ventral pressure was higher than dorsal in only two cases (*S. mokarran* and *S. zygaena*).

**Figure 4:**
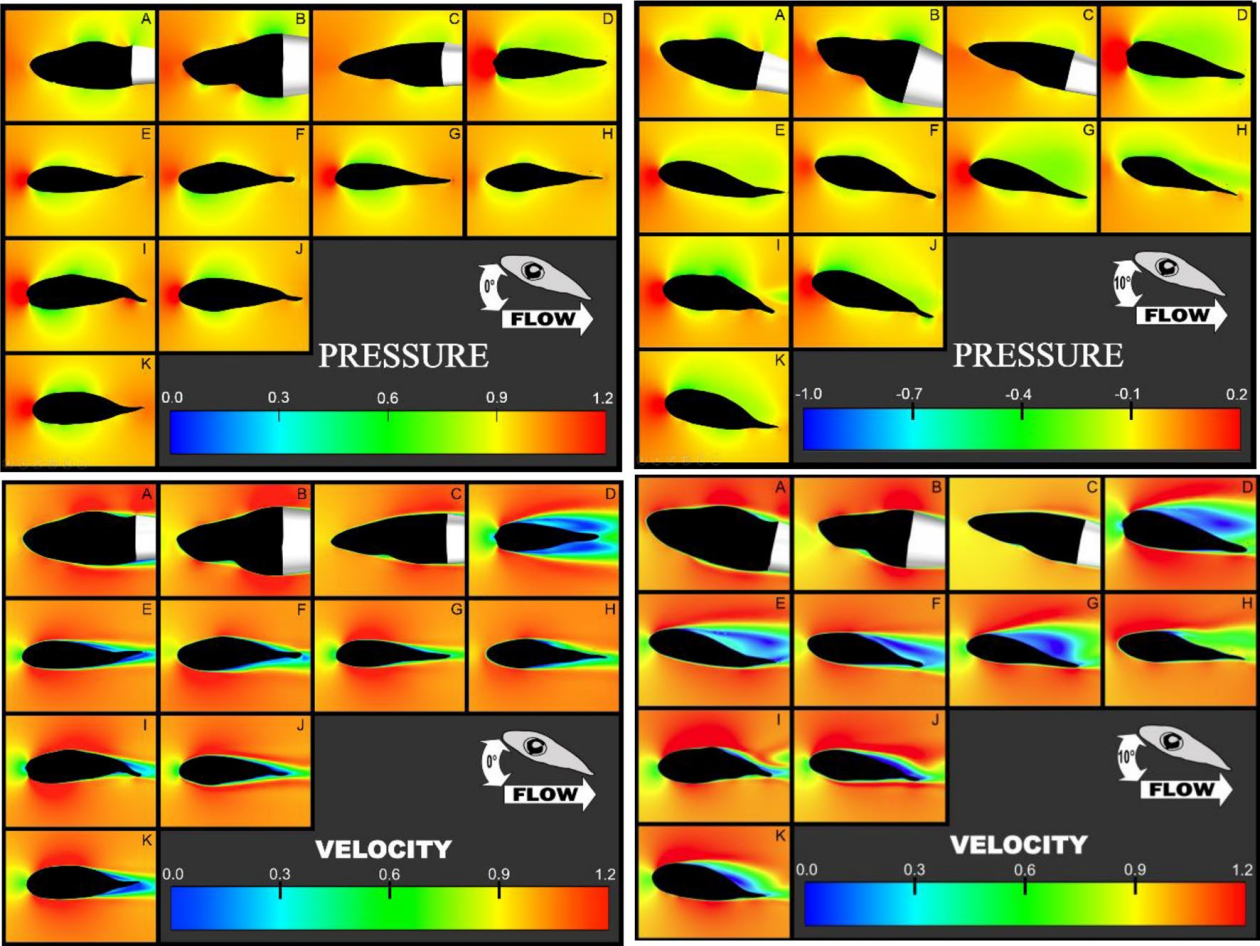
Composite of *distal* pressure clipping planes at α = 0° (top left) and α  = 10° (top right) and velocity clipping planes at α = 0° (bottom left) and α = 10° (bottom right) from all eleven species. Note the interfamilial difference in both magnitude of the gradient and posterior edge flow separation at α = 10°, but the lack thereof at the hypothesized α = 0°; (**A**) *C. leuca*s, (**B**) *N. brevirostris*, (**C**) *C. limbatus*, (**D**) *E. blochii*, (**E**) *S. corona*, (**F**) *S. media*, (**G**) *S. tudes*, (**H**) *S. tiburo*, (**I**) *S. lewini*, (**J**) *S. zygaena*, (**K**) *S. mokarran.*

Planar sections taken parasagittally through the fluid domain from the dorsal to ventral side of the cephalophoil further clarified patterns of fluid pressure (Fig. 4). At α = 0°, no substantial wing-like pressure gradient between dorsal and ventral surfaces was apparent medially or distally in any species. Only at positive angles (e.g. α = 10°) did sphyrnid head shapes generally produce larger pressure gradients than carcharhinids. This occurred both medially and distally and was particularly clear in the region just ahead of the leading edge of the cephalofoil where a high-pressure stagnation point was observed. In sphyrnids, green-colored regions of the fluid domain occurred atop the head (indicating lower pressures), whereas in carcharhinid species, this effect was very much reduced. Fluid velocity clipping planes taken at α = 0° through the same regions showed corroborative patterns (Fig. 4). No dorsoventral velocity gradient was noted in any group except at positive α.

**Figure 5:**
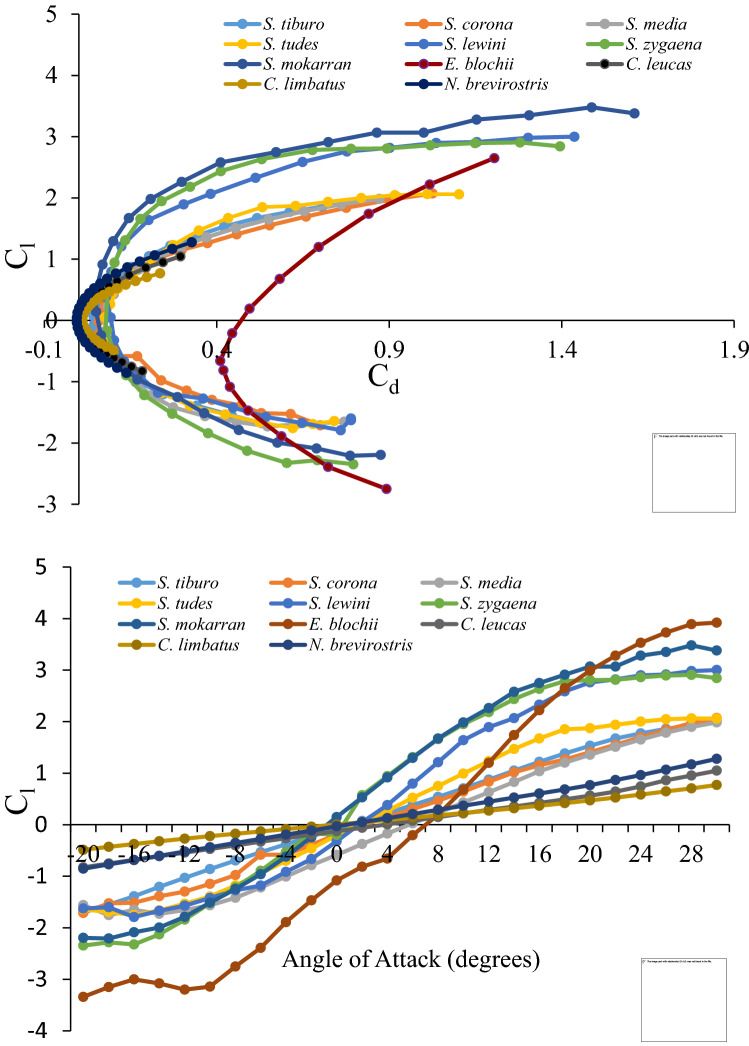
Lift coefficient (C_l_) by drag coefficient (C_d_) (**A**) and angle of attack (**B**) (− 20° to + 30°) for all species. Note the grouping of species along the x-axis by both family and cephalofoil size.

**Figure 6:**
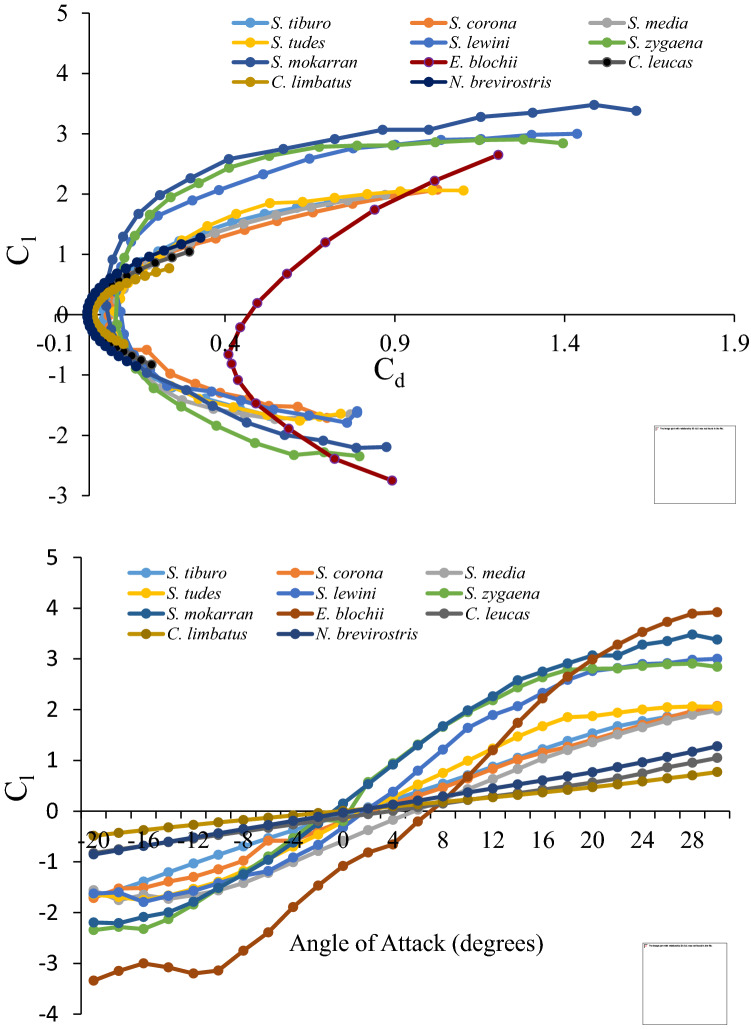
Drag coefficient (Cd) plotted against the angle of attack.

Cross-taxonomic comparison of drag polars plotted for all species (Fig. 5A) indicated greater overall drag values in sphyrnid head morphologies. At α = 0°, drag coefficients ranged from a low of 0.0423 for *S. tiburo* to a high of 0.4379 for *E. blochii* and, interfamilially, drag decreased with decreasing cephalofoil size. Drag also increased with increasing α at a much greater rate in sphyrnids. At α = 0°, lift coefficients for sphyrnids (Fig. 5B) ranged from a low of − 1.0815 for *E. blochii* to a high of − 0.077 for *S. tiburo*. Lift coefficients for Carcharhinid species were essentially zero (0.004 to − 0.0083) at α = 0°. Only *S. mokarran* and *S. zygaena* cephalofoils showed positive lift (0.146 and 0.1996, respectively) at α = 0°. The lift-to-drag ratio was conspicuously lower across all attack angles for the winghead shark, (*E. blochii*) than for other shark species. We likewise examined the relationship between drag coefficient and angle of attack (Fig. 6). At the same angle of attack, Carcharhinid sharks were found to have the lowest lift and drag values, followed by Sphyrnid sharks with small and then intermediate sized cephalofoils. *Eusphyra blochii* possessing the largest cephalofoil was distinctly different demonstrating significantly larger drag coefficients and showing the greatest change in lift as attack angle changed.

**Figure 7:**
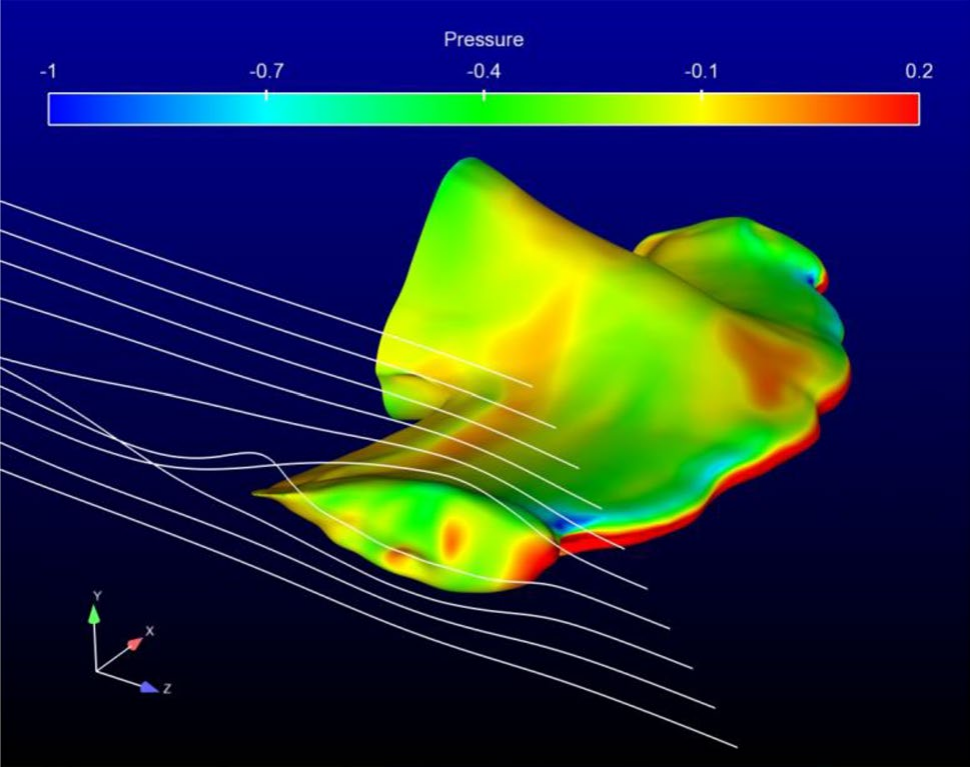
Pressure isosurface of *S. lewini* shown from a right lateral aspect (α = 0°)*.* Fluid streamlines were generated from a particle trace. Note the resemblance to wingtip vortex generation at the distal tip of the cephalofoil.

Computational fluid dynamic post-processing included a basic check for “wingtip” vortex formation in hammerheads at the distal ends of the cephalofoil. Streamlines generated from a particle trace depicted vorticity in some species (such as *S. lewini*) at positive angles of attack (Fig. 7).

## Discussion

There have been various hypotheses directed toward explaining the function of the hammerhead cephalofoil. In this study, we examined the suggestions that this structure provides a hydrodynamic advantage by increasing maneuverability and by producing lift similar to a cambered wing. Our analysis revealed several important points that cast light on these ideas: (1) Although some regions of high and low pressure were visible at a zero attack angle, when we examined surface pressure contours, there was a lack of an overall net dorso-ventral pressure gradient. (2) Mean pressure differences across dorsal and ventral surfaces of the cephalofoil were typically small and, in most cases, were higher on the dorsal surface. (3) Pressure and velocity cutting planes did not indicate significant pressure or velocity differentials between dorsal and ventral surfaces except at some angle of inclination with respect to flow. This was true even in *distal* regions where (as naturalists and biologists have previously suggested) in parasagittal section the structure’s profile most closely bears superficial resemblance to a man-made cambered wing. (4) Lift coefficients at α = 0° were negative for all but two species. Taken together, these results do not support a cambered wing hypothesis.

Although our results suggest that significant lift is not produced during horizontal swimming in Sphyrnids, the generation of lift at positive angles of attack during routine swimming can nevertheless be important. For example, the Leopard shark, *Triakis semifasciatus* and the Bamboo shark, *Chiloscylium punctatum*, held the body at + 11 and 9°, respectively, during horizontal, steady swimming in a swim tunnel, to counteract the antero-ventral reaction force created by the heterocercal caudal^22^. However, this does not appear to be the case for Sphyrnids. In many observations of in-situ shark swimming by one of the authors (GRP), hammerhead sharks are often observed swimming parallel to flow and often in close proximity to a featureless, horizontal substrate when foraging for prey.

To provide additional insight regarding potential hydrodynamic function, we examined lift and drag across a wide range of attack angles for all species. Modern day, man-made, cambered foils are generally typified by C-shaped, parabolic drag polars; drag values tending to increase concurrently with lift values as attack angle becomes more extreme. Drag polars depicted in this study, indicated that drag typically increased at a faster rate than lift. This effect is imparted by boundary layer separation at the trailing edge of the foil progressing increasingly toward its center. This continues until flow separation ultimately occurs to such a great extent that the lifting efficiency (the ratio of lift to drag) is undermined, and the foil stalls^23^. Lift polars typically exhibit an overall positive linear slope progressing to a maximum (the stall point), inflecting suddenly, and taking on a sharp downward trajectory thereafter.

In previous work, no notable differences were observed across the drag polars of sphyrnid species, and little evidence of wing-like hydrodynamic properties of the cephalofoil among small sphyrnids was cited^11^. In contrast, we observed substantial differentiation across sphyrnid species with regard to both drag *and* lift polars as well as substantial interfamilial differences. In our examination of lift and drag coefficients, we observed a pattern whereby curves were grouped broadly by slope. These groups corresponded with sharks featuring discretely different head morphologies: (1) carcharhinids, (2) hammerheads possessing small cephalofoils, i.e., *tudes, media, tiburo* and *corona*, (3) hammerheads with intermediate cephalofoils, i.e. *mokarran*, *zygaena* and *lewini*, and (4) a large cephalofoil (*E. blochii)*. Across species, slope tended to decrease with decreasing aspect ratio of the head. The above groupings precisely mirror phylogenetic groupings as determined by morphological, isozyme and mtDNA sequence data^24^.

Despite its common name (winghead shark) the *E. blochii* cephalofoil generated the greatest amount of drag and produced, at low angles of attack, the least amount of lift. It may be noteworthy that the *E. blochii* head morphology displayed the greatest rate of change in lift coefficient as attack angle changed. Additionally, at the highest attack angles, the lift coefficients were the greatest of any species. The results for this species likewise do not support the cambered wing lift hypothesis. However, the relatively rapid change in lift generated at positive attack angles implies that the *E. blochii* cephalofoil in particular may provide a hydrodynamic advantage via an increase in maneuverability. It may be significant that the *E. blochii* diet was found to consist of about 93% teleost fishes, apparently of the family Clupeidae^25^, whereas other hammerhead species feed predominantly on stingrays, crabs, and other bottom-dwelling organisms^26^. The predominance of highly mobile clupeids in the diet of *E. blochii* may reflect the greater mobility that its cephalofoil provides.

The increased maneuverability hypothesis can be interpreted in concert with the hydrodynamic data generated as part of this study. Relevant to this study are findings regarding greatly enhanced hypaxial musculature in hammerheads relative to carcharhinid (typical) sharks^10^. This musculature may indicate the importance of the head in facilitating prey capture by generating rapid shifts in trajectory. As the results presented here show, if the head were depressed to the maximum possible extent indicated in the aforementioned study (− 15°), the reaction force produced would be substantial. In larger sphyrnids, the downward moment produced would necessarily be large (owing to the large surface area of the cephalofoil). In smaller sharks, a change in trajectory might be facilitated just as easily using a smaller cephalofoil (thus lessening the tradeoff between cephalofoil utility and its associated drag) given their smaller overall mass.

Previous research has concluded that the cephalofoil likely has a negative effect on stability^27^. Its position at the far anterior end of the shark does increase its mechanical advantage substantially, and our results confirm that the magnitudes of the reaction forces produced increase rapidly as attack angle deviates from level. The inference here is that the cephalofoil may serve as a forward rudder under active control of hypaxial and epaxial musculature, thus providing for rapid dives and ascents. It should be noted that the cephalofoil may actually confer stability during turning, as it was observed that sphyrnid sharks did not roll during sharp turns, as did their carcharhinid counterparts^28^. Prey detection, via an increased number of Ampullae of Lorenzini, and prey capture do seem to be the prevailing directions in current thought regarding cephalofoil function. Larger hammerhead species are known to prey disproportionately on skates and rays, and increased maneuverability could confer an advantage in avoiding such prey defenses. We caution, however, that it remains uncertain from our results and other literature presently available whether or not the structure provides a prey-capture advantage via increased maneuverability.

The drag coefficients estimated in this study can be used to make ecophysiological comparisons between hammerhead and typical carcharhinid sharks. An avenue for making these comparisons is to examine cephalofoil drag coefficients across species at the same lift coefficients (*C*_*L*_ = 0.2). Relative to the cephalofoil of other hammerhead species, the *E. blochii* cephalofoil generated 6.4 × (*S. corona*) to 12.6 × (*S. lewini*) greater drag. Additonally, the *E. blochii* cephalofoil generated 22.6 × , 32.3 × , and 39.7 × greater drag when compared with *C. limbatus*, *C. leucas*, and *N.* brevirostris, respectively. Finally, comparing drag coefficients of sharks possessing typical head morphologies with the remaining hammerhead species revealed that the cephalofoil generated 1.9 × (*S. tiburo*—*C. limbatus* comparison) and 6.2 × (*S. corona*—*N. brevirostris* comparison) increased drag.

An important implication of the increased drag in hammerheads is the concurrent increase in energy expenditure necessary to maintain forward motion. This is especially relevant in obligate ram-ventilating sharks such as hammerheads. During level, unaccelerated cruising, sufficient force (i.e. thrust) must be imparted to a body to overcome the resistive force of drag and move that body through a fluid. In this instance, the required thrust is equal to the drag force^23^. Calculation of the thrust required can be easily accomplished by the equation:

$$T_{R} = \, D \, = 1/2 \, \rho v^{2} AC_{D}$$

where *T*_*R*_ is required thrust, *D* is the drag force, *ρ* is the fluid density (1.023026 × 10^3^ kg/m^3^ for seawater at 25 °C and 35 ppt salinity), *υ* is the fluid velocity (held constant at 1 m/s), *C*_*D*_ is drag coefficient, and *A* is the respective planform area (outline of an area as seen from above) of each head model.

Thrust required for forward motion requires power generation and hence energy expenditure on the part of the fish. Using data obtained from CFD and the above equation, it is possible to calculate and compare the difference in drag force (and corresponding thrust required) for differing shark head morphologies. For these calculations we chose to compare adult *S. lewini* and *C. limbatus* because Reynolds’ numbers were comparable (1.25 × 10^5^ and 1.32 × 10^5^, respectively). Thus, for *S. lewini* and *C. limbatus* a drag force of approximately 9.34 and 1.007 Newtons, respectively was calculated. Conversion from Newtons to pounds-force (where 1 N = 0.2248 *lb*_*f*_) yields 2.099 and 0.226 *lbf* for *S. lewini* and *C. limbatus,* respectively.

Hence, our calculations indicate that, possessing a cephalofoil requires an almost 10 × increase in thrust for *S. lewini* compared with a similarly sized *C. limbatus.* It is noteworthy that the drag difference in this example is conservative since *S. lewini* has a higher reference length and higher Reynolds number. It would be anticipated that the greater thrust and energy necessary for *S. lewini* to swim at the same speed as *C. limbatus* would result in increased food consumption to offset increased metabolism and, potentially, a cascade of physiological changes that would accompany those increases. However, various compensating mechanisms could offset the increased energy requirement of possessing a cephalofoil. For instance, hammerhead sharks may reduce cruising speeds, enhance static lift mechanisms, and/or possess a more efficient metabolism. In actuality, a general trend toward higher metabolic rates has been shown in ram-ventilating sharks^15,29^. Sphyrnid metabolic rates are typically high as well, with rates as high as 168 mg O_2_ kg^−1^ h^−1^ in *S. tiburo* and 189 mg O_2_ kg^−1^ h^−1^ in *S. lewini*^30–32^*.* Thus, it seems unlikely that sphyrnids offset drag-related energetic loss via lower metabolism. To the contrary, metabolic rates are higher in sphyrnids (which may exacerbate the problem of drag-related energy loss).

An interesting behavior has been observed in *S. mokarran*^33^ which may be relative to the results presented herein. These sharks have been reported to swim *in-situ* on their sides up to 90% of the time. This change in orientation during swimming may provide increased lift via the repositioning of the large dorsal fin, which is estimated to reduce the energetic cost of swimming by approximately 10%^33^. While it is not known if this behavior is widespread across Sphyrnids, hammerheads in general possess relatively large dorsal fins (relative to other shark species) and adopting this swimming behavior could be related to possession of a hydrodynamically costly cephalofoil.

There was limited evidence to support the suggestion that the cephalofoil produces trailing vortices similar to those generated by aircraft wings^11^. Wing tip vortices, also called lift-induced vortices, result from air flowing from below the wing where pressure is high, around the tip and onto the top of the wing where pressure is low, in a circular motion. The absence of significant trailing vortices observed during this study at zero angle of attack reflects the absence of pressure differences between dorsal and ventral surfaces of the cephalofoil. However, to better understand cephalofoil vorticity, time-accurate simulations should be used to view flow patterns across a temporal gradient. The possibility exists that the energetic cost of possessing a cephalofoil may be offset by increased swimming efficiency through active flow control. Interaction of the cephalofoil with appropriately placed downstream appendages, such as the hammerhead pectoral fins, may significantly transform flow ahead of the caudal fin and cause beneficial interactions with the caudal. An examination of the potential for these interactions between the cephalofoil and fins, as well as how changes in orientation of the fins relative to the cephalofoil during undulatory swimming might alter this interaction, would necessitate CFD analysis of the entire hammerhead body during active swimming which was beyond the scope of this study.

The results of this study suggest that the hammerhead cephalofoil functions as a foil insofar as it operates as a symmetric foil, or thin plate requiring alteration of its attack angle for the production of lift. It does not appear to possess sufficient camber to generate lift at α = 0. Our analysis suggests that the possession of a cephalofoil may increase maneuverability. In light of evidence presented regarding active control of cephalofoil attack angle via hypaxial and epaxial musculature^10^, we suggest that this structure may function as a forward rudder (and perhaps as a fluid dynamic brake) at the anterior end of the animal facilitating more rapid changes in position in the water column and increased maneuverability during the final moments of prey capture. It is important to recognize that the Spalart–Allmaras turbulence model^21^ can reasonably predict drag and lift at small to moderate angles of attack, but may not be well-suited for high angles of attack. However, this would not alter our observation that there was little to no significant lift generated at zero attack angle. It also should not invalidate the potential relative differences in maneuverability observed in this study between species at low to moderate angles of attack. Relative to this is the observation that electrically stimulated *S. lewini* have been observed to maximally depress and elevate their cephalofoil ca 15° and 30°, respectively. Anecdotal observations in situ by one of the authors (GRP) reveals that these sharks rarely flex their cephalofoil to the maximum extent during routine swimming. Interestingly, these data suggest that hammerhead sharks may pay a greater energetic price for their head morphology when compared with typical sharks. However, the hydrodynamic price of possessing a cephalofoil, may be offset by the increase in prey detection and capture that this unusual structure may provide. Finally, in the face of an almost complete absence of information regarding the hydrodynamic role of the cephalofoil, and the fact that no computational fluid dynamics study of fish swimming existed prior to this work, we suggest that this research be viewed as a first approximation of the hydrodynamic and energetic costs and benefits of possessing a cephalofoil. Ultimately, we hope that it will stimulate additional research using CFD in the biological sciences and, in particular, to study fish swimming hydrodynamics.

## Data Availability

All data is available through the University of Mississippi Institutional Data Repository.
